# Association of APOE genotype and ELOVL2 methylation with brain function in non‐demented adults during aging

**DOI:** 10.1002/alz.092733

**Published:** 2025-01-09

**Authors:** Natalya V Ponomareva, Tatiana V Andreeva, Irina L Kuznetsova, Maria Protasova, Svetlana Kunizheva, Daria Kupryanova, Rodion Konovalov, Marina V. Krotenkova, Ekaterina P Kolesnikova, Daria Malina, Elena V. Kanavets, Vitaly Fokin, Sergey N Illarioshkin, Evgeny I Rogaev

**Affiliations:** ^1^ Research Center of Neurology, Moscow Russian Federation; ^2^ Vavilov Institute of General Genetics of Russian Academy of Sciences, Moscow Russian Federation; ^3^ Vavilov Institute of General Genetics of Russia Academy of Sciences, Moscow Russian Federation; ^4^ Vavilov Institute of General Genetics of Russian Academy of Sciences, Moscow Russian Federation; ^5^ Center for Genetics and Life Science, Sirius University of Science and Technology, Sochi Russian Federation; ^6^ Department of Psychiatry, Umass Chan Medical School, Shrewsbury, MA USA; ^7^ Center for Genetics and Life Science, Sirius University of Science and Technology, Sochi Russian Federation

## Abstract

**Background:**

The ε4 allele of the apolipoprotein E (APOE4+) genotype and aging synergistically contribute to the risk of Alzheimer's disease (AD), but the mechanisms underlying their influence are not completely understood. The methylation of ELOVL2 DNA accounts for 70% of the variance in the aging epigenetic clock. The ELOVL2 gene is essential for synthesizing long polyunsaturated fatty acids, crucial for cell membrane integrity, inflammation modulation, and energy maintenance. This study investigated the impact of APOE genotype and ELOVL2 methylation on EEG alpha rhythm and functional MRI (fMRI) resting‐state functional connectivity (rsFC) of brain networks in non‐demented adults during aging.

**Method:**

We examined EEG alpha sub‐bands power and individual alpha peak frequency (IAPF) and the fMRI rsFC in 151 non‐demented volunteers, age range 20‐84 years, stratified by APOE genotype. APOE ε3/ε3 subgroup (APOE4‐) included 104 subjects, APOE ε4/ε3 (APOE4+) subgroup – 47 subjects. ELOVL2 cg16867657 methylation was examined in the blood in subgroup of 56 individuals (35 APOE4‐ and 21 APOE4+). The individuals underwent fMRI tsFC of the brain using the CONN Matlab/SPM‐based toolbox (Whitfield‐Gabrieli and Nieto‐Castanon, 2012). Informed written consent was obtained from all participants. All subjects underwent a neurological examination and cognitive screening.

**Result:**

The presence of the APOE4+ genotype was associated with more pronounced alpha rhythm slowing and more pronounced decrease of fMRI rsFC during aging. Age‐related increase of ELOVL2 methylation was observed. The partial correlation analysis, with age as a covariate, revealed a significant correlation between ELOVL2 methylation and IAPF.

**Conclusion:**

More pronounced alpha rhythm slowing and fMRI rsFC reduction in APOE4+ carriers during aging can be linked to synaptic dysfunction and deterioration of white matter integrity. Impaired lipid metabolism, driven by age‐dependent ELOVL2 methylation, may accelerate the impact of the APOE4+ genotype on neurophysiological alterations during aging. These changes increase the risk of AD developed.

**Funding**:

This study was supported by Russian Science Foundation (Project No. 19‐75‐30039 to TA genotyping; Project No. 22‐15‐00448 to NP, RK, VF, EK EEG and fMRI analysis and MP for genotyping) and by Ministry of Science and Higher Education of the Russian Federation (Agreement 075‐10‐2021‐093 project GEN‐RND‐2017).

